# Energy Spectral Behaviors of Communication Networks of Open-Source Communities

**DOI:** 10.1371/journal.pone.0128251

**Published:** 2015-06-05

**Authors:** Jianmei Yang, Huijie Yang, Hao Liao, Jiangtao Wang, Jinqun Zeng

**Affiliations:** 1 School of Business Administration, South China University of Technology, Guangzhou, P. R. China; 2 Business School, University of Shanghai for Science and Technology, Shanghai, P. R. China; 3 Guangdong Province Key Laboratory of Popular High Performance Computers, College of Computer Science and Software Engineering, Shenzhen University, Shenzhen, P. R. China; 4 Department of Physics, University of Fribourg, Fribourg, Switzerland; University of Michigan, UNITED STATES

## Abstract

Large-scale online collaborative production activities in open-source communities must be accompanied by large-scale communication activities. Nowadays, the production activities of open-source communities, especially their communication activities, have been more and more concerned. Take CodePlex C # community for example, this paper constructs the complex network models of 12 periods of communication structures of the community based on real data; then discusses the basic concepts of quantum mapping of complex networks, and points out that the purpose of the mapping is to study the structures of complex networks according to the idea of quantum mechanism in studying the structures of large molecules; finally, according to this idea, analyzes and compares the fractal features of the spectra in different quantum mappings of the networks, and concludes that there are multiple self-similarity and criticality in the communication structures of the community. In addition, this paper discusses the insights and application conditions of different quantum mappings in revealing the characteristics of the structures. The proposed quantum mapping method can also be applied to the structural studies of other large-scale organizations.

## Introduction

With the development of internet, open-source communities (OSC) and their derivatives, crowd-source communities, are becoming new forms to produce knowledge [1–6]. In past years many things about OSC, including their production patterns, the motivation and behavior of participants and so on, were well studied [7–9]. And the complex network models [10] have become increasingly important for studying the production structure of OSC [11,12] and structures of other online communities [13–16].

As collective activities of human society, the knowledge production activities must be accompanied by large-scale communication activities. Behaviors of communication activities will be greatly helpful for us to understand the dynamical mechanisms of complex systems. The structures of communication activities (communication structures for short) can also be described with complex networks, in which the nodes are the communicators and the edges are the communication activities shared by the pairs of nodes. Now in the academic circle of complex networks there are a few researches on the communication structures [17]. In the present work, models of complex networks based on different quantum mappings are used to find nontrivial behaviors from empirical records of communication activities.

Yang et al. proposed a mapping from complex networks to quantum systems [18–20]. Suppose the adjacent matrix of a complex network with *N* identical nodes is *A*, whose elements *A*_*ij*_ = 1 or 0 if the nodes *i* and *j* are connected or disconnected, respectively. They first regard the complex network as a large molecule and map the adjacent matrix *A* to Hamiltonian of the large molecule; and then detect the structural symmetry of the complex network by analyzing scale invariance in the eigenvalue sequence of matrix *A*.

In this paper we first construct the complex network models of 12 periods of communication structures of CodePlex C # community based on large-scale data collections. Then we refine the concept of quantum mapping of a complex network; give the classification of the mapping, reveal that the quantum mapping in [18–20] is merely the simplest “structural mapping”; and define corresponding Hamiltonians and energy spectra of a complex network in different mappings. Afterwards we discuss why the quantum mapping of complex networks is necessary and point out the purpose of the mapping is to study the structural symmetry and symmetry breaking of networks according to the idea in quantum mechanism. Finally, following this idea, we analyze and compare the spectral fractal features and their changes over time in different quantum mappings of the communication networks of the community, and from the perspective of symmetry reveal the specific characteristics of the communication structures.

Our research reveals that in different quantum mappings the energy spectra of any communication network of the community all have multifractality (i.e. multiple self-similarity), long-range correlation and some other specific features. The features have nothing to do with the mapping ways of the networks so they are the intrinsic attributes of the communication structures. Moreover, from the perspective of communication, these attributes show the community is in the critical state that is the most efficient state of complex systems [21].

However, there are also large differences in the multifractal degree of the spectra among different mappings of any communication network of the community. The “structural mapping” just roughly reflects the spectral fractal features and slowly reflects their changes over time. Therefore it is not enough just using “structural mapping” to deeply understand the structure of networks.

## Materials and Methods

### Data

CodePlex community (http://www.codeplex.com) built up by Microsoft in 2006 is an open-souse community. Microsoft does not claim ownership of the materials (http://www.codeplex.com/site/legal/terms) (S1 File). So the data collection was in compliance with the terms and conditions of CodePlex website. In this paper, the C# sub-community (called community for short), the most important sub-community in CodePlex, is taken as the study object.

To every project in the community there is a “discussions” webpage for publishing opinions on. Every speech of communicators on the webpage of a project is kept as a communication record. To every project there also is a “change list” webpage to record the coding activities of producers for the project. Every coding activity, i.e. production, is kept on the webpage of the project as a production record.

LocoySpider (a data acquisition software) is taken as the tool to collect the records from May 2006 to July 2012 of the community. The collected data contain a total of 144.342 thousand communication records, and a total of 198.616 thousands production records, including 2,136 projects (software modules) and 3,233 producers.

Further, from the communication records we get the data of communication intensity between each pair of communicators in 12 periods (half year as the period, see S1 Data). From the production records we get the numbers of production times of communicators in 12 periods (S2 Data). The process to get a certain communicator’s number of production times in a period is as follows. First from the production records find out his/her number of production times for every project in this period, then sum up the numbers for all projects, and the sum is to be his/her number of production times in this period.

The data are preprocessed via cleaning. For example, if some project has not its “discussion” webpage or there is no content in the webpage, the data of this project will be deleted.

### Communication Networks

We build 12 models of communication networks for Codeplex C# community based on the data (S1 and S2 Data) from the second half of the year 2006 to the first half of the year 2012. Each communication network corresponds to a period of half a year.

The steps to build the communication network to a period are as follows. Firstly every communicator is taken as a node and the weight of the node equals to the number of production times of the communicator. If two communicators have speech records on the “discussions” webpage of same project, connect them with an edge. Secondly, the numbers of speech times of different communicators about one project are usually different. Suppose for project *A* communicator *1* has *m1* times of speech while communicator *2 m2*, then we take the smaller number between *m1* and *m2* as the communication intensity of communicator *1* and *2* for project *A*. In a same period communicator *1* and *2* may publish speeches about many projects of the community, so we take the sum of their communication intensities for all these projects as the total communication intensity of communicator *1* and *2*, i.e. the weight of the edge between them.

For communication network of each period, the size (it refers to the number of nodes or edges), the number of connected graphs (CGs), the size of the maximum connected graph (MCG) and the percentage of the MCG size with respect to the total are shown in Table 1. After calculation, it is shown that the sum of edge weights for a node (it is different from the node weight defined in this paper) of every network follows power-law distributions. The exponents of the accumulative distributions are from 1.01 to 2.00. The sum values for every network also show differences in magnitude.

**Table 1:** Sizes of communication networks, Numbers of CG and Sizes of MCG.

Period	Number of nodes (edges)of the network	Number of CG	Number of nodes (edges)of MCG	the percentage of the MCG nodes(edges) with respect to the total
second half of 06	653(2032)	71	278(1268)	0.4257(0.6240)
first half of 07	1206(6747)	157	454(3644)	0.3765(0.5401)
second half of 07	1562(5986)	271	567(3084)	0.3630(0.5152)
first half of 08	2064(7562)	370	910(5252)	0.4409(0.6945)
second half of 08	2323(7625)	400	1135(5793)	0.4886(0.7597)
first half of 09	3180(10477)	569	1621(7927)	0.5097(0.7566)
second half of 09	3667(11445)	714	1877(8309)	0.5119(0.7260)
first half of 10	3975(13039)	768	2109(10122)	0.5306(0.7763)
second half of 10	4019(14967)	791	2151(11914)	0.5352(0.7960)
first half of 11	4125(14690)	833	2458(13261)	0.5959(0.9027)
second half of 11	3667(10960)	789	1755(7506)	0.4786(0.6849)
first half of 12	3741(9619)	857	2028(8196)	0.5421(0.8521)

Furthermore the communication network in the first half of the year 2011 is taken as an example to analyze CG. The network has a total of 4125 nodes, 14,690 edges and 833 CGs. The node size of 833 CGs follows a power-law distribution, and the exponent of accumulative distribution is 1.94. Its MCG has 2,458 nodes, 13,261 edges; the proportion of nodes accounts for 59.6%, and the proportion of the edges accounts for 90.3%. (S1 Fig) This shows that the MCG of a communication network contains the basic connection relations of the network and can reflect its structural characteristics.

### Quantum mapping of complex networks

This paper extends the concept of quantum mapping of complex networks in references [18–20]. Here the quantum mapping not only refers to mapping nodes of a network to atoms in a large molecule and the edges to the chemical bonds between the atoms, but also refers to mapping an edge weight that reflects the connection intensity of a pair of nodes to the hopping energy required for an electron to jump between the atoms, and the node weight that reflects node attributes to the energy of the electron on the atom site to which it belongs, consequently a complex network is mapped to a large molecule.

Furthermore, based on the idea of Hückel model for the molecular system [19,22], which points out electrons in a molecule are mainly influenced by the potential of the atoms that binds the electrons, other potentials of atoms can be taken as a small perturbation, and the interactions between the electrons can be ignored. Thereby, the wave function of one molecule can be regarded approximately as the linear combination of the wave functions of isolated atoms.

The quantum mapping method of a complex network is very useful and promising to understand the network’s structure. Because after quantum mapping, a complex network can be regarded as a large molecule; therefore through the Hamiltonian and energy spectrum (i.e. eigenvalue sequence of Hamiltonian) of the large molecule, we can define the Hamiltonian and energy spectrum of the network. Here we call network’s Hamiltonian as the energy matrix of the network. In this way the theory and method about the energy spectrum in quantum mechanism can be used to reveal the structural characteristics of the network. For example, the fractal behavior of the energy spectrum of a network suggests us the network has the quasi-periodic structure and is in the critical state between the order state and disorder state [23,24].

Based on above-mentioned concept of quantum mapping, we classify the quantum mapping of complex networks into three ways. In the first way referred to as "comprehensive mapping” (CM), nodes and edges (i.e. structure), edge weights and node weights are all mapped in accordance with above defined rules. In the second way referred to as "intermediate mapping” (IM), without the node weights just the structure and edge weights are mapped because the node weights are considered to be the same. In the third way referred to as "structural mapping”(SM), only the structure is mapped, node weights and edge weights are excluded for the same reason denoted in "intermediate mapping”.

The complex network model for a real-world problem is often called the weighted network in which there are different weights among nodes and different weights among edges. But according to the research needs, its quantum mapping can be in three ways: “comprehensive mapping”, “intermediate mapping” and “structural mapping”. In different ways the network can be mapped to different large molecules and then corresponds to different Hamiltonians.

In the theory of Network Science there are other two kinds of complex network models: edge-weighted network and Boolean network. In the light of their definitions, the edge-weighted network has two mappings: “intermediate mapping” and “structural mapping”; and Boolean network has only one mapping way: “structural mapping”.

### Hamiltonians of a complex network in different mappings

The Hamiltonian of Hückel model of molecular systems in Dirac operator form [19,22] is shown in Eq (1). Where *εi* stands for the energy of the electron on site *i* and if the molecule contains more than one species of atom, the *ε*_*i*_ will be different for different species; *t*_*ij*_ refers to the energy depending on the species of the atom *i* and *j* that the electron is hopping between; <*ij*> serves to tell us that the sum is only over those pairs of atoms joined by a chemical bond. Dirac operator|*i*><*j*| corresponds to the *N*-order matrix in which element of row *i* and column *j* is 1 and all other elements are 0s; whereas $\sum_{i=1}^{N}\varepsilon_{i}\left|i\rangle \langle i\right|$ is the *N*-order diagonal matrix with diagonal elements as *ε*_*i*_.

$$\hat{H}=\sum_{i=1}^{N}\varepsilon_{i}\left|i\rangle \langle i\right|+\sum_{\langle ij\rangle }t_{ij}\left|i\rangle \langle j\right|$$ (1)

Suppose that the adjacency matrix of a node-weighted and edge-weighted complex network with *N* nodes is *A = (A*_*ij*_ *)*_*N × N*_, where *A*_*ij*_ describes the connection between nodes i and j of the network. It is easy to see that *A*_*ij*_ has the same effect as <*ij*> that describes the connection between atoms *i* and *j* in Eq (1). So, we use A_*ij*_ to replace <*ij*> in (1) and obtain Hamiltonian of the network based on Hückel model.

In the first quantum mapping way, because not only the structure but also node weights and edge weights all are mapped, Hamiltonian of the network is:

$$\hat{H}=\sum_{i=1}^{N}\varepsilon_{i}\left|i\rangle \langle i\right|+\sum_{i\neq j}^{N}A_{ij}t_{ij}\left|i\rangle \langle j\right|$$ (2)

Where *ε*_*i*_ is represented by the weight of node *i*, because the weight of node *i* denotes the energy that depends on location *i* of the network; and *A*_*ij*_ *t*_*ij*_ is represented by the weight of edge *ij*, because the weight of edge *ij* denotes the energy that depends on the interaction intensity between node *i* and *j*.

In the second quantum mapping way, we consider structure and edge weights and don’t consider node weights, so *ε*_*i*_ corresponding to the weight of node *i* in Eq (2) can be regarded as 0, and the Hamiltonian is

$$\hat{H}=\sum_{i\neq j}^{N}A_{ij}t_{ij}\left|i\rangle \langle j\right|$$ (3)

In the third quantum mapping way, we only consider the connection relations of nodes, *ε*_*i*_ in Eq (2) can be treated as 0 and *t*_*ij*_ related to the weight of edge *ij* can be treated as 1, and the Hamiltonian is

$$\hat{H}=A$$ (4)

In summary, the Hamiltonians of a node-weighted and edge-weighted network in three quantum mappings have three forms, see Eqs (2), (3) and (4); correspondingly, the network has three energy spectra. In addition, it can also be known that the Hamiltonians of an edge-weighted network have two forms, see Eqs (3) and (4); while the Hamiltonian of Boolean network has only form (4).

It should be pointed out that from the arithmetic rules on Dirac operator, the mathematical form of Hamiltonian of a complex network is completely the same as that of related algebraic matrix of the network. For example, in "structural mapping", the Hamiltonian of a node-weighted and edge-weighted network is just the same as its adjacency matrix. However, the Hamiltonian of a network is no longer abstract matrix, but the energy matrix, in which each number has specific energy meaning. Furthermore, the eigenvalues of Hamiltonian of the network show the magnitude of energy that the network may have.

### Hamiltonians of communication networks

After the discussion on Hamiltonians of general complex networks, now we discuss Hamiltonians of the communication networks. The communication networks of 12 periods of the community are node-weighted and edge-weighted networks, therefore they all have three quantum mappings. In the first way of “comprehensive mapping”, the Hamiltonian of a communication network to a period has been shown in Eq (2).

Here the weight of node *i*, namely ε_*i*_, is represented by the number of production times of communicator *i* in this period, for the number reflects the communicator’s energy that relates to his/her location in the network; The weight of edge *ij*, namely *A*_*ij*_ *t*_*ij*_, is represented by the communication intensity between communicators *i* and *j* in this period, for the communication intensity shows the communication energy between communicators *i* and *j*.

In the second way of "intermediate mapping" and third way of "structural mapping", the Hamiltonians of communication network to a period are represented by Eqs (3) and (4) respectively. The meanings of Hamiltonian elements are the same as those of Hamiltonian elements in the "comprehensive mapping".

Further, we should point out that: First, in the present work, we construct the network model from records in specified time duration, map it to a large molecule in a certain way and get consequently its Hamiltonian. This procedure implies that the system in the time duration, i.e., a snapshot of the total records, is described with a steady state, in which the Hamiltonian is independent explicitly with time (the energy keeps constant) and the wave function oscillates periodically. From records for successive time durations we can construct complex networks, which form a series of state snapshots. The time-dependence of Hamiltonian is described by the evolution of the nodes and links in the series.

Second, summation of all the interactions between the nodes is a measure of energy of the system in macroscopic time scale, corresponds to the average level of communication. However, the microscopic states of the system are changing rapidly. Measurement of energy is actually an average procedure of the energies for microscopic states. Each egenvector of the Hamiltonian is a possible microscopic state of the system, while the corresponding egenvalue is the energy (communication level) of the microscopic state. Perturbations from environment will induce transitions between the microscopic states. The transition probability between each pair of microscopic states is proportional to the strength of perturbation, while it is inversely proportional to the spacing between the two corresponding energy levels. Hence, energy spacing is an essential measure to quantify transition probabilities between two microscopic states. This kind of transition means changes of contributions of nodes to the communication level.

### Method for Analyzing Energy Spectra

Once the Hamiltonians of communication networks are obtained, the energy spectra of the networks can be easily calculated. One of the core topics in quantum mechanics is about the behaviors of energy spectra. From the perspective of symmetry, the spectral multifractality needs to be analyzed firstly. Because the MCG of a communication network contains the basic connection relations of the network, we focus on the spectral multifractality of the MCG. Regarding energy spectra as time series [25,26], the behaviors are analyzed by using Multifractal detrended fluctuation approach (MF-DFA) [27].

In MF-DFA, if the series {*x*_1_,*x*_1_,…,*x*_*N*_} (i.e. the energy spectrum {*E*_1_ ≤ *E*_2_ ≤ ≤ *E*_*N*_} of MCG in this paper) is long-range power-law correlated, the q-order fluctuation function *F*_*q*_ *(s)* increases, for large values of scale *s*, as a power-law

$$F_{q}\left(s\right)={\left\{\frac{1}{2N_{s}}\sum_{v=1}^{2N_{s}}{\left[F^{2}\left(s,v\right)\right]}^{q/2}\right\}}^{1/q}∼s^{h\left(q\right)}$$ (5)

where *N*_*s*_ *≡ int(N/s)*, nonoverlapping segments of equal length *s*, *s* is the length of the segment, i.e. the scale of measurement, *F* ^*2*^(*s*,*v*) is the variance for segment *v* of the series with the segment local trend by a least-square fit.

If the fluctuation of a series is measured with different scales, the measurement results of the fluctuation would be different. The fractal series in this paper refers to that the series has self-similarity. Therefore to judge a series is or isn't fractal we should observe whether or not the measurement results of the fluctuation follow the same law under different scales. The practical method of the judgment is to analyze whether or not there exists a power law relation between *F*_*q*_(*s*) and *s*, where *F*_*q*_(*s*) is mean value of the *q*-order fluctuations of *2N*_*s*_ segments under a certain scale *s*. The reason of using *q*-order fluctuation function is to respectively survey the self-similarity features of the large fluctuation subsets and small fluctuation subsets of the series.

For monofractal series, *h*(*q*) is independent of *q*, for multifactal series, *h*(*q*) is correlated with *q*. When *q* > 0, *F*_*q*_(*s*) describes the scaling behavior of the segments with large fluctuations, when *q* < 0, *F*_*q*_(*s*) describes that of the segments with small fluctuations. The *h(2)* of the stationary series is the Hurst exponent, so *h(q)* is also regarded as the *q*-order Hurst exponent.

We know that the multifractality is often showed by singularity strength (multifractal scaling index) *a* and singularity spectrum (multifractal spectrum function) *f*(*a*). Based on the standard partition function *Z*_*q*_(*s*) *q*-order Mass exponent *τ*(*q*) [27,28] in Eq (6) and Legendre transform in Eq (7), the relationship of *h(q)* with *α* and that of *h(q)* with *f(α)* could be derived as (8)

$$Z_{q}\left(s\right)=\sum_{v=1}^{N/s}{\left|Y\left(vs\right)-Y\left[\left(v-1\right)s\right]\right|}^{q}∼\;s^{\tau \left(q\right)},\tau \left(q\right)=qh\left(q\right)-1$$ (6)

$$\alpha =\tau^{\prime }\left(q\right),f\left(\alpha \right)=q\alpha -\tau \left(q\right)$$ (7)

$$\alpha =h\left(q\right)+qh^{\prime }\left(q\right),f\left(\alpha \right)=q\left[\alpha -h\left(q\right)\right]+1$$ (8)

It should be noted that *f*(*a*) actually is the exponent of power-law relationship between the counts of subsets *N*(*s*) and scale s:*N*(*s*) ∼ s^-*f*(*a*)^ [28–31], so it is the fractal dimension of the subset whose singularity strength is *α*. The maximum value of *f*(*a*) is the fractal dimension of the subset having largest count (it is approximately seen as the fractal dimension of the set). It can be known from *f*’(*a*) = *q*, *f*(*a*) reaches to its maximum when *q* = 0. In addition, the common generalized fractal dimension *D*(*q*) is defined by the partition function, and when *q* = 0 the value of partition function is equal to “the result of count”, so *D(0)* also represents the fractal dimension of the set [28]. MF-DFA requires that the series is of compact support and only determines the positive *h*(*q*), when *h*(*q*) is close to zero, where amended method needs to be adopted [27].

## Results

**Fig 1:**
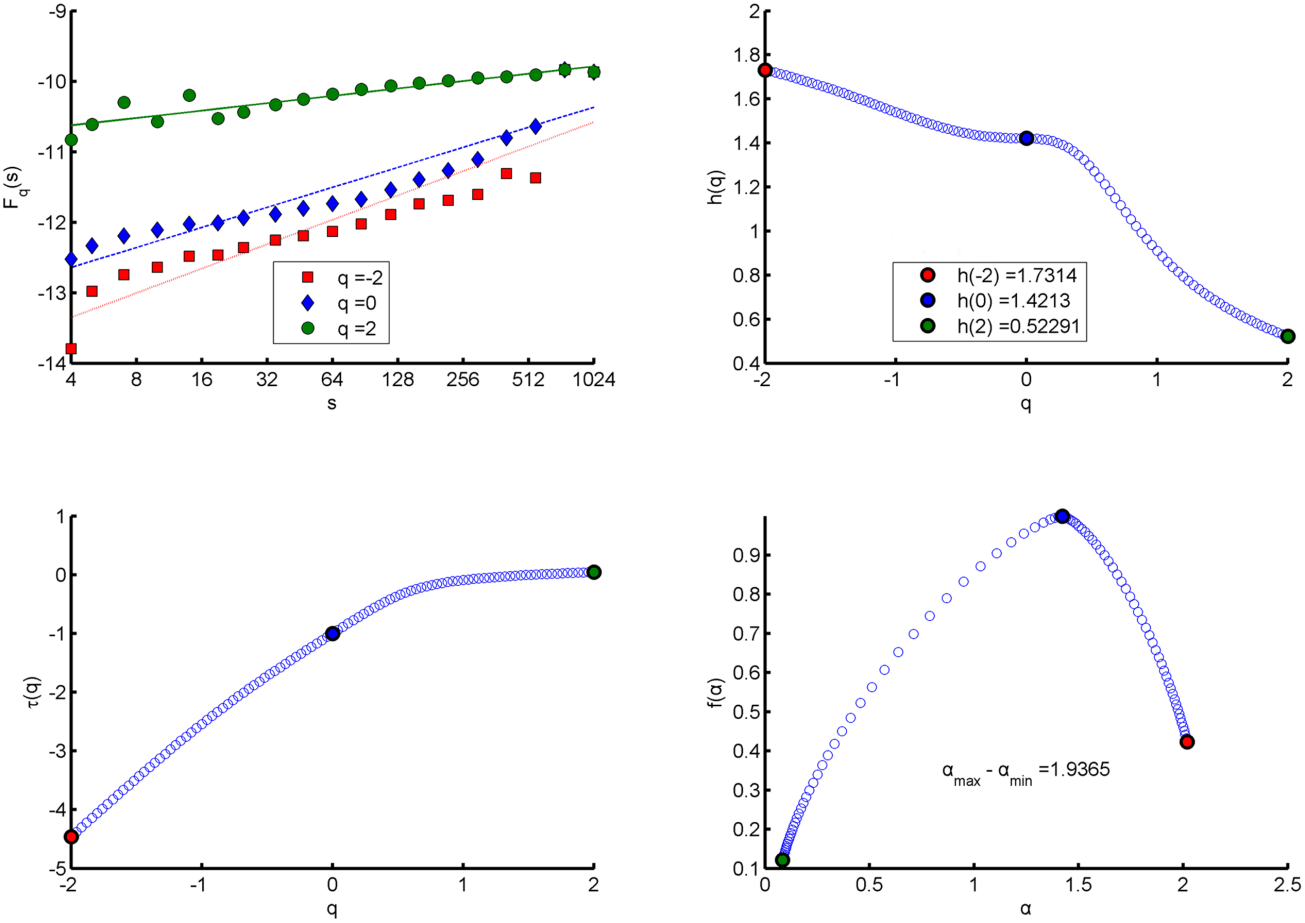
Energy Spectral fractal features in “comprehensive mapping” of the communication network for first half of the year 2010. (a) q-order Fluctuation function *F*_*q*_(*s*) versus scale s in log-log plots. (b) q-order Hurst exponent *h*(*q*). (c) q-order Mass exponent *τ*(*q*). (d) Multifractal spectrum function *f*(*α*).

**Fig 2:**
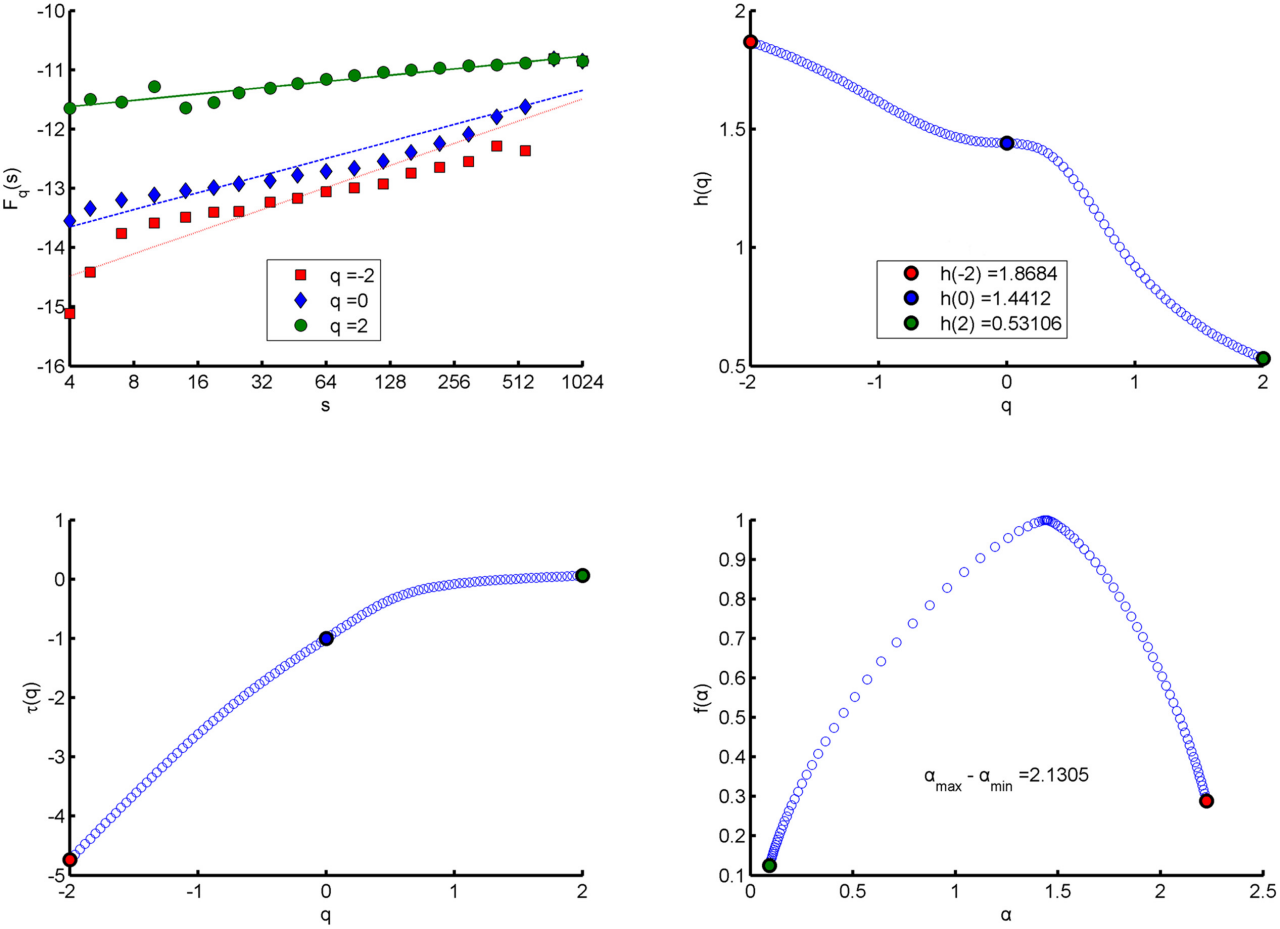
Energy Spectral fractal features in “intermediate mapping” of the communication network for first half of the year 2010. (a) q-order Fluctuation function *F*_*q*_(*s*) versus scale s in log-log plots. (b) q-order Hurst exponent *h*(*q*). (c) q-order Mass exponent *τ*(*q*). (d) Multifractal spectrum function *f*(*α*).

**Fig 3:**
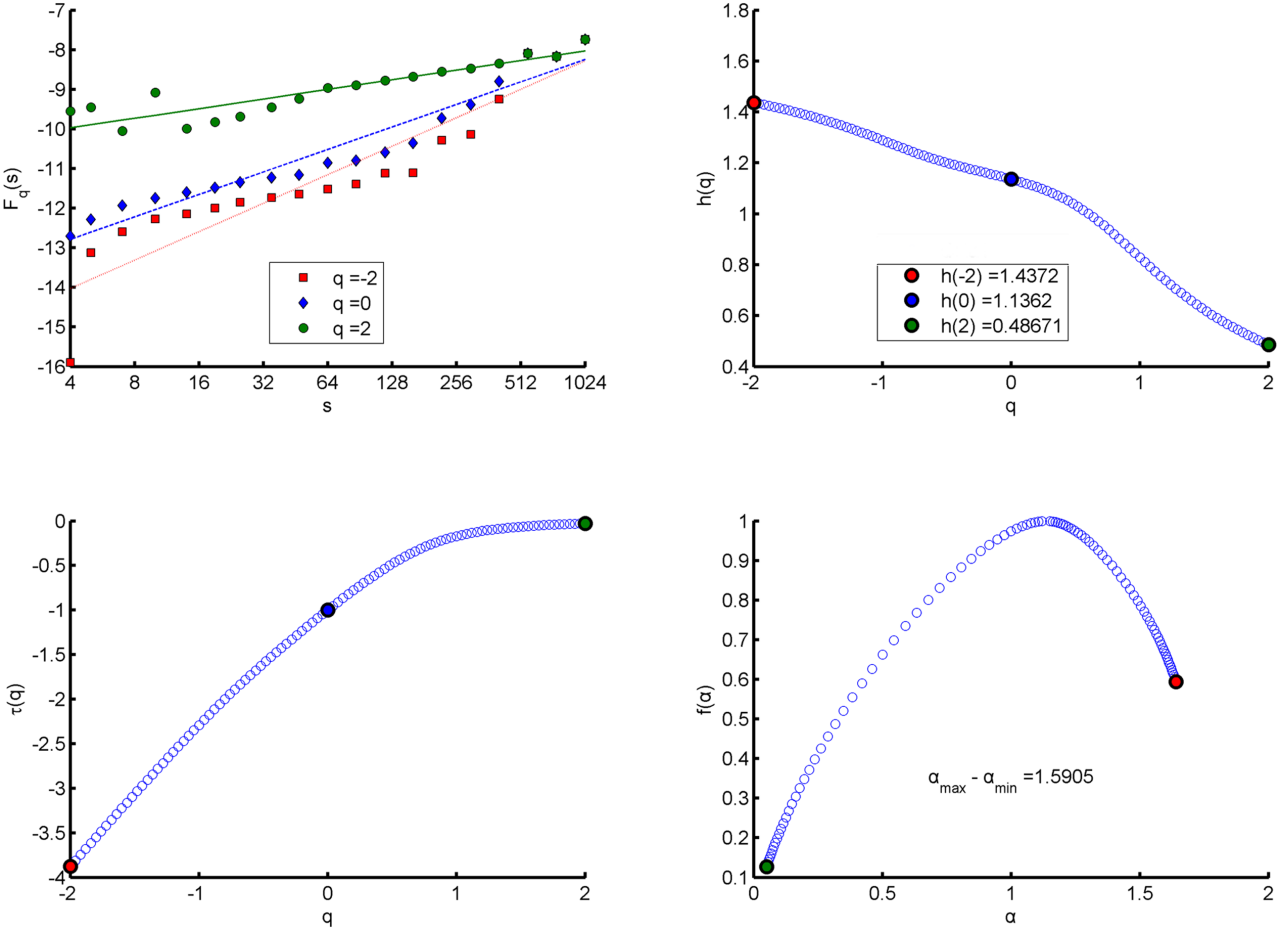
Energy Spectral fractal features in “struture mapping” of the communication network for first half of the year 2010. (a) q-order Fluctuation function *F*_*q*_(*s*) versus scale s in log-log plots. (b) q-order Hurst exponent *h*(*q*). (c) q-order Mass exponent *τ*(*q*). (d) Multifractal spectrum function *f*(*α*).

Firstly for the MCG of the communication network to each period, calculate the energy spectrum *E = {E*_*1*_ *≤E*_*2*_ *≤⋯≤E*_*N*_ *}* of Hamiltonian in each mapping way designed in 2.4. Then take the spectrum after removing repeated roots as the initial series *{x*_*1*_,*x*_*2*_,*⋯x*_*j*_ *⋯*,*x*_*K*_ *}*, and calculate the fractal indicators of the series by using MF-DFA. The calculation results are shown in Table 2 and Figs 1–3. Italics in Table 2 indicate that the symmetric value range of q of the network to this period is between *-1*.*5* and *+1*.*5*, so we cannot calculate energy spectral Hurst exponents for corresponding networks. The similarities and differences of fractal features of energy spectra in different mappings can be found from Table 2 and Figs 1–3.

**Table 2:** Fractal indicators of energy spectra in different mappings of the communication networks for 12 periods.

		q = -2 *(q = -1*.*5)*			q = 0			q = 2 *(q = 1*.*5)*				
Period	MW	h(q)	α	f(α)	h(q)	α	f(α)	h(q)	α	f(α)	Δα	Δf(α)
SH of 2006	CM	2.1168	2.4405	0.3525	1.7176	1.7124	1	0.7418	0.2794	0.0752	2.1611	0.2773
	IM	4.2230	4.5714	0.3031	3.6439	3.6246	1	2.4355	1.9624	0.0539	2.6090	0.2492
	SM	1.7516	2.0016	0.5000	1.3466	1.3346	1	0.5741	0.0979	0.0477	1.9037	0.4523
*FH of 2007*	*CM*	*1*.*9800*	*2*.*1356*	*0*.*7667*	*1*.*7750*	*1*.*7660*	*1*	*0*.*7414*	*0*.*1131*	*0*.*0576*	*1*.*8390*	*0*.*7091*
	*IM*	*2*.*2246*	*2*.*5000*	*0*.*5870*	*1*.*7975*	*1*.*7865*	*1*	*0*.*7293*	*0*.*0885*	*0*.*0387*	*2*.*4115*	*0*.*5483*
	*SM*	*1*.*6386*	*1*.*8066*	*0*.*7479*	*1*.*4017*	*1*.*3978*	*1*	*1*.*0772*	*0*.*7196*	*0*.*4635*	*1*.*0871*	*0*.*2844*
SH of 2007	CM	3.0201	3.2779	0.4843	1.9467	1.9046	1	0.6433	0.1626	0.0386	*1*.*8974*	0.4457
	IM	3.0337	3.3104	0.4467	1.9615	1.9268	1	0.8580	0.3922	0.0685	2.9182	0.3782
	SM	1.5818	1.7524	0.6587	1.2529	1.2396	1	0.5938	0.2454	0.3032	1.5070	0.3555
FH of 2008	CM	1.7798	2.0531	0.4535	1.5376	1.5379	1	0.5633	0.1140	0.1014	1.9391	0.3521
	IM	2.0140	2.3184	0.3911	1.5653	1.5627	1	0.5380	0.0927	0.1093	2.2257	0.2818
	SM	1.4257	1.5822	0.6869	1.1821	1.1752	1	0.5237	0.0916	0.1357	1.4907	0.5512
*SH of 2008*	*CM*	*2*.*6211*	*2*.*9537*	*0*.*5012*	*1*.*8584*	*1*.*8623*	*1*	*0*.*9933*	*0*.*3646*	*0*.*0570*	*2*.*5891*	*0*.*4442*
	*IM*	*2*.*6716*	*3*.*0513*	*0*.*4303*	*1*.*8648*	*1*.*8662*	*1*	*0*.*6441*	*0*.*0011*	*0*.*0356*	*3*.*0502*	*0*.*3947*
	*SM*	*1*.*2137*	*1*.*3539*	*0*.*7897*	*1*.*0644*	*1*.*0603*	*1*	*0*.*5978*	*0*.*1518*	*0*.*3311*	*1*.*2021*	*0*.*4586*
*FH of 2009*	*CM*	*2*.*7774*	*3*.*0668*	*0*.*5659*	*2*.*5424*	*2*.*5432*	*1*	*1*.*3077*	*0*.*6505*	*0*.*0143*	*2*.*4162*	*0*.*5516*
	*IM*	*3*.*0410*	*3*.*3859*	*0*.*4828*	*2*.*6652*	*2*.*6603*	*1*	*1*.*7125*	*1*.*1418*	*0*.*1440*	*2*.*244*	*0*.*3388*
	*SM*	*3*.*1980*	*3*.*4398*	*0*.*6373*	*2*.*8214*	*2*.*8090*	*1*	*1*.*7669*	*1*.*1095*	*0*.*01391*	*2*.*3303*	*0*.*62339*
*SH of 2009*	*CM*	*1*.*7858*	*1*.*9280*	*0*.*7867*	*1*.*6965*	*1*.*6993*	*1*	*0*.*7024*	*0*.*07840*	*0*.*0640*	*1*.*8496*	*0*.*7227*
	*IM*	*2*.*7225*	*3*.*3030*	*0*.*5386*	*2*.*4339*	*2*.*4326*	*1*	*1*.*1884*	*0*.*5531*	*0*.*0470*	*2*.*4771*	*0*.*4916*
	*SM*	*1*.*2430*	*1*.*4450*	*0*.*6969*	*1*.*0691*	*1*.*0656*	*1*	*0*.*6197*	*0*.*1484*	*0*.*2930*	*1*.*2967*	*0*.*4039*
FH of 2010	CM	1.7314	2.0200	0.4229	1.4213	1.4207	1	0.5229	0.0835	0.1211	1.9365	0.3018
	IM	1.8684	2.2240	0.2887	1.4412	1.4403	1	0.5311	0.0935	0.1249	2.1305	0.1638
	SM	1.4372	1.6404	0.5936	1.1362	1.1307	1	0.4867	0.0499	0.1265	1.5905	0.4671
SH of 2010	CM	2.4925	2.7648	0.4554	1.7263	1.7112	1	0.6100	0.1402	0.0639	2.6246	0.3915
	IM	1.6914	1.8829	0.6170	1.5873	1.5947	1	0.6900	0.2381	0.0962	1.6449	0.5208
	SM	1.3182	1.4014	0.8336	1.2043	1.1987	1	0.5805	0.1798	0.1986	1.2216	0.635
FH of 2011	CM	1.5480	1.8006	0.4949	1.4066	1.4126	1	0.5863	0.1710	0.1694	1.6296	0.3255
	IM	1.6276	1.9453	0.3639	1.4404	1.4479	1	0.6062	0.1816	0.1508	1.7636	0.2131
	SM	1.3805	1.5654	0.6302	1.1183	1.1136	1	0.5334	0.1082	0.1496	1.4572	0.4806
SH of 2011	CM	1.8538	2.1595	0.3886	1.4704	1.4662	1	0.5383	0.1129	0.1492	2.0466	0.2394
	IM	1.8693	2.1842	0.3703	1.4784	1.4757	1	0.6112	0.1971	0.1717	1.9871	0.1986
	SM	1.3213	1.5312	0.5801	1.0891	1.0864	1	0.6951	0.3250	0.2599	1.2062	0.3202
FH of 2012	CM	1.8038	2.1123	0.3830	1.4703	1.4683	1	0.4884	0.0006	0.0244	2.1117	0.3586
	IM	2.6545	3.0338	0.2414	1.4996	1.4963	1	0.5267	0.0510	0.0486	2.9828	0.1928
	SM	2.1932	2.4136	0.5592	1.1091	1.1039	1	0.6709	0.3129	0.2840	2.1008	0.2752

MW: mapping way. FH: first half. SH: second half.

### The q-order fluctuation function *F*_*q*_ *(s)* and the q-order Hurst exponent *h(q)*

Seen from the values of *F*_*q*_(*s*) and *h*(*q*) in Table 2 and Figs 1–3, the fractal features of energy spectra in three mappings have the following similarities. Firstly, no matter what *q* is equal to in its value range, the values of *F*_*q*_(*s*) increase with the scale *s*; and when *q* is a fixed value, the growth rate *h*(*q*) of *F*_*q*_(*s*) versus scale *s* in log-log plot remains unchanged, which means that energy spectra of networks have fractal property. Secondly, *F*_*q*_(*s*) with different *q* corresponds to different value of *h*(*q*), which means that the energy spectra have multifractal property. Finally, because the *F*_*q*_(*s*) with large (positive) *q* describes the scaling behavior of subsets with large fluctuations of a spectrum and the *F*_*q*_(*s*) with small (negative) *q* describes the behavior of subsets with small fluctuations; the fact that the value of *h*(*q*) decreases when *q* increases suggests the growth rates for small fluctuation subsets are greater than those for large fluctuation subsets.

### The Hurst exponent

For stationary series, *h(2)* is identical to the Hurst exponent. Almost all Hurst exponents of energy spectra of the networks are greater than 0.5, which indicates that the energy spectra in three mappings have features of long-range correlation. But the degree of long-range correlation of energy spectra in “structural mapping” is the weakest and stable over time. For the “structural mapping”, “comprehensive mapping” and “intermediate mapping”, the means of the Hurst exponents in all periods are 0.58767, 0.6483 and 0.6372, respectively.

### Singularity strength *α* and width of singular spectrum *Δα*

In MF- DFA, *α* represents the change rate of Mass exponent *τ*(*q*) versus *q*; and in standard partition function-based multifractal formalism, it is the change rate of density *p*(*s*) versus scale *s* in log-log plot [28]. The *Δα* is the difference between the maximum singularity strength and the minimum one of a spectrum in the value range of *q*, which indicates the multifractal degree.

The results show that energy spectra in three mappings all satisfy that singularity strengths of large fluctuation subsets (*q* > 0) are weaker than those of small fluctuation subsets (*q* < 0). And in three mappings of a network, on average for 12 periods, the singularity strength *α* when *q* = 0 in “structural mapping” is the minimum and that in “intermediate mapping” is the maximum. Similarly, on average for 12 periods, the width of singular spectrum *Δα* in “structural mapping” is also the minimum and the mean is 1.5328 (variance 0.1523), the *Δα* in “intermediate mapping” is also the maximum with the mean being 2.3704 (variance 0.2130), while the *Δα* in “comprehensive mapping” is slightly smaller than that in “intermediate mapping” with the mean being 2.0867 (variance 0.0968). Moreover, except slight decrease over time in “comprehensive mapping”, the widths in other two mappings are stable.

### Multifractal spectrum function *f (α)* and *Δf (α)*

In the symmetrical value range of *q*, the curves *f(α)* of energy spectra of networks in three mappings show right-hook shapes (see Figs 1d–3d), that is to say, the values of *f(α)* when *α* terminates at the right end of the curves are larger than the values of *f(α)* when *α* terminates at the left end. The *α* values at the right end correspond to *q* < 0 and the *α* values at the left end correspond to *q* > 0, so right- hook shape shows that the decay rate of *f(α)* along with *q* increasing when *q* > 0 is faster than the decay rate along with *q* decreasing when *q* < 0. Our tests also show that *f(α)* values corresponding to *q* < -10 at the right end are still positive, but when *q* > +2, *f(α)* values at the left end corresponding to some periods are negative. Further, the deep meaning of right- hook shapes is that the value range of *q* corresponding to the part with small fluctuations of an energy spectrum is wider; there are greater differences in singularity strengths and fractal dimensions among its subsets with small fluctuations. So we can say that there are stronger multifractality in the part with small fluctuations of the spectrum than in the part with large fluctuations. And for the series of compact support, *f(α)* obtains the maximum value 1 when *q* = 0 (*D*_*0*_ = 1).

The *Δf(α)* is the difference between the maximum *f(α)* and the minimum *f(α)* and represents the right hook degree of a spectrum. The greater *Δf(α)* is the stronger the right-hook degree is. The mean of the values of *Δf(α)* for 12 periods in “intermediate mapping” is the minimum (0.3376, variance 0.0170) and that in “structural mapping” is the maximum (0.4423, variance 0.0146). In addition, the values of *Δf(α)* in “structural mapping” are stable over time, and in other two mappings all show a slight decline.

For clarity, the above findings about the similarities and differences are summarized in Table 3.

**Table 3:** Summary of the similarities and differences of fractal features in different mappings.

MW	MF	RH	h(q)	h(2)(Hurst exponent)		α (in the value range of q)	α(q = 0)	Δα	f(α) (in the value ranges of q)	Δf(α) (Degree of right hook)
CM	√	√	subsets with small fluctuations are larger	almost all>0.5	max	subsets with small fluctuations are larger	mid	middle	subsets with small fluctuations are wider	middle
	√	√	ditto	ditto	decline slightly	ditto	mid	stable	ditto	decline slightly
IM	√	√	subsets with small fluctuations are larger	almost all>0.5	middle	subsets with small fluctuations are larger	max	max	subsets with small fluctuations are wider	min
	√	√	ditto	ditto	decline slightly	ditto	max	decline slightly	ditto	decline slightly
SM	√	√	subsets with small fluctuations are larger	almost all>0.5	min	subsets with small fluctuations are larger	min	min	subsets with small fluctuations are wider	max
	√	√	ditto	ditto	stable	ditto	min	stable	ditto	stable

MW: mapping way. MF: Multifractality. RH: right hook

## Conclusions and Discussions

### Conclusions concerning the study object

From above findings about the similarities in the three mappings, it can be summarized that the energy spectra of any communication network of this community have features of multifractality and long-range correlation. Moreover, there are more different singularities among the small fluctuation subsets than among the large ones. The growth rates (in log-log plot of fluctuation functions with respect to scale) and the singularity strengths of the small fluctuation subsets are greater than those of the large ones. The energy spectral fractal indicators of these networks are relatively stable or show slightly declining trend from the second half of the year 2006 to the first half of the year 2012.

However, the energy spectra of any communication network in different mappings also have different features: Firstly, the long-range correlation in “structural mapping” is the minimum while that in “comprehensive mapping” is the maximum. Secondly, the singularity strength of the subset having largest count (*q* = 0) in “structural mapping” is the minimum while that in intermediate mapping” is the maximum. Thirdly, the width of singular spectrum in “structural mapping” is also the minimum while in “intermediate mapping” is the maximum. Fourthly, the right-hook degree in “structural mapping” is the maximum while that in “intermediate mapping” is the minimum. Fifthly, all the fractal indicators in “structural mapping” are very stable over time, while those in other two mappings show slightly downward trend.

In conclusion, on one hand, all energy spectra of communication networks have some common fractal features, regardless of mapping ways and time, which indicates that they are the intrinsic attributes of the communication structures modeled by the networks. And from the implied meaning of spectral fractal of networks, we can conclude that the communication structures have the features of multiple self-similarity and criticality. On the other hand, the values of the fractal indices of any communication network in three mappings are quite different.

### Conclusions concerning research methods

In the three mappings of communication networks, the “structural mapping” only considers whether there is a connection relationship between a pair of nodes. The “intermediate mapping” also considers the intensity of the relationship based on “structural mapping”, and the “comprehensive mapping” further considers node attributes based on “intermediate mapping”. It could be known from conclusions about the study object that the energy spectrum in “structural mapping” of a communication network generally has the fractal features of energy spectra in other two mappings of the network, so for simplicity, one can directly use it to analyze the fractal features of the network structure.

However, compared with using “comprehensive mapping” and “intermediate mapping”, the energy spectral multifactuality of a network revealed by using “structural mapping” is weaker, the right hook feature is stronger and the fractal indices are stable over time, which means that the “structural mapping” just roughly and slowly reflects network’s fractal features and their changes as time goes by. Therefore, to deeply understand the structure of networks we need to use “comprehensive mapping” or “intermediate mapping”.

We think the following problems need to be discussed.

### The value range of *q*

Under the condition that singularity strength α and fractal dimension *f (α)* are greater than 0, the multifractal range of an energy spectrum could be represented by the symmetrical value range of *q*. And corresponding *f(α)* curve shows the fractal feature of right or left hook. In this paper, several tests about the symmetrical value range of *q* are conducted. It is found that when values of *q* are from -2 to 2, the values of *α* and *f(α)* are greater than 0 for most periods. The symmetrical value range of *q* is determined by the value of *q* corresponding to the value of *f(α)* that decreases to zero first at left or right end. Of course, the fractal features of energy spectra could also be studied by taking the value range of *q* asymmetrically.

### About scale *s* >1

In the standard partition function-based multifractal formalism (*p*_*i*_(*s*)∼*s* ^*ai*^ where *p*_*i*_(*s*) denotes density of subset *i*), when *s* tends to zero, the scale exponent *a*_*i*_ is defined as singularity strength of subset *i* [28]. Because *s* is less than 1, the value of *a*_*i*_ of the subset *i* having higher density should be smaller. In MF-DFA, the scale *s* is greater than 1; it can be known that the large fluctuation corresponds to the high density for stationary and normalized series [27]. Therefore, the value of *a*_*i*_ of the subset *i* with larger fluctuation should be larger, but the case calculation in this paper shows the result that the larger fluctuation subset corresponds to smaller *ai* value.

Careful analysis shows that the Mass index *τ(q)* calculated from the actual data in this paper is one upward convex function versus *q*, so the larger *q* is, the smaller the derivative of *τ(q)* is. And *α* is the derivative of *τ(q)* versus *q*, thus the conclusion is obtained that *a*_*i*_ of the subset *i* with larger fluctuation must be smaller. Therefore, *p*_*i*_(*s*) ∼ *s* ^*ai*^ in the standard partition function-based multifractal formalism needs to be revised as *p*_*i*_(*s*) ∼ *s* ^*-ai*^ if one defines *a*_*i*_ of this paper in terms of *p*_*i*_(*s*).

### The repeated roots

MF-DFA requires series of compact support, i.e. *x*_*j*_ = 0 for an insignificant fraction. When there are repeated roots in eigenvalues, some segments of the series will be composed entirely of the same data when the scale becomes small. The differences between the data and their local trend will be 0s, and the detrended series does not meet the condition of compact support. So we remove the repeated roots during calculation.

## Concluding Remarks

This paper reveals the characteristics of communication structures of the OSC by comparing the energy spectral fractal behaviors in different quantum mappings of the communication networks. The proposed quantum mapping method can also be applied to the structural study of other large-scale online communities.

It should be noted that it is only a study from the perspective of symmetry based on quantum mapping for the communication structures of OSC, and further study is needed from the perspective of symmetry breaking. And the study also is needed to be done on whether the conclusions concerning different quantum mappings of the communication networks can be extended to general complex networks.

## Supporting Information

S1 Data Communication intensities of communicators in 12 periods. (RAR)

S2 Data Numbers of production times of communicators in 12 periods. (RAR)

S1 Fig MCG of the communication network of first half of the year 2011. (PDF)

S1 File CodePlex Terms of Use. (DOCX)
