# Supplementary figures and images for: Energy Spectral Behaviors of Communication Networks of Open-Source Communities
Source: PLoS One. 2015 Jun 5;10(6):e0128251. doi: 10.1371/journal.pone.0128251 (PMC4457875; doi:10.1371/journal.pone.0128251)

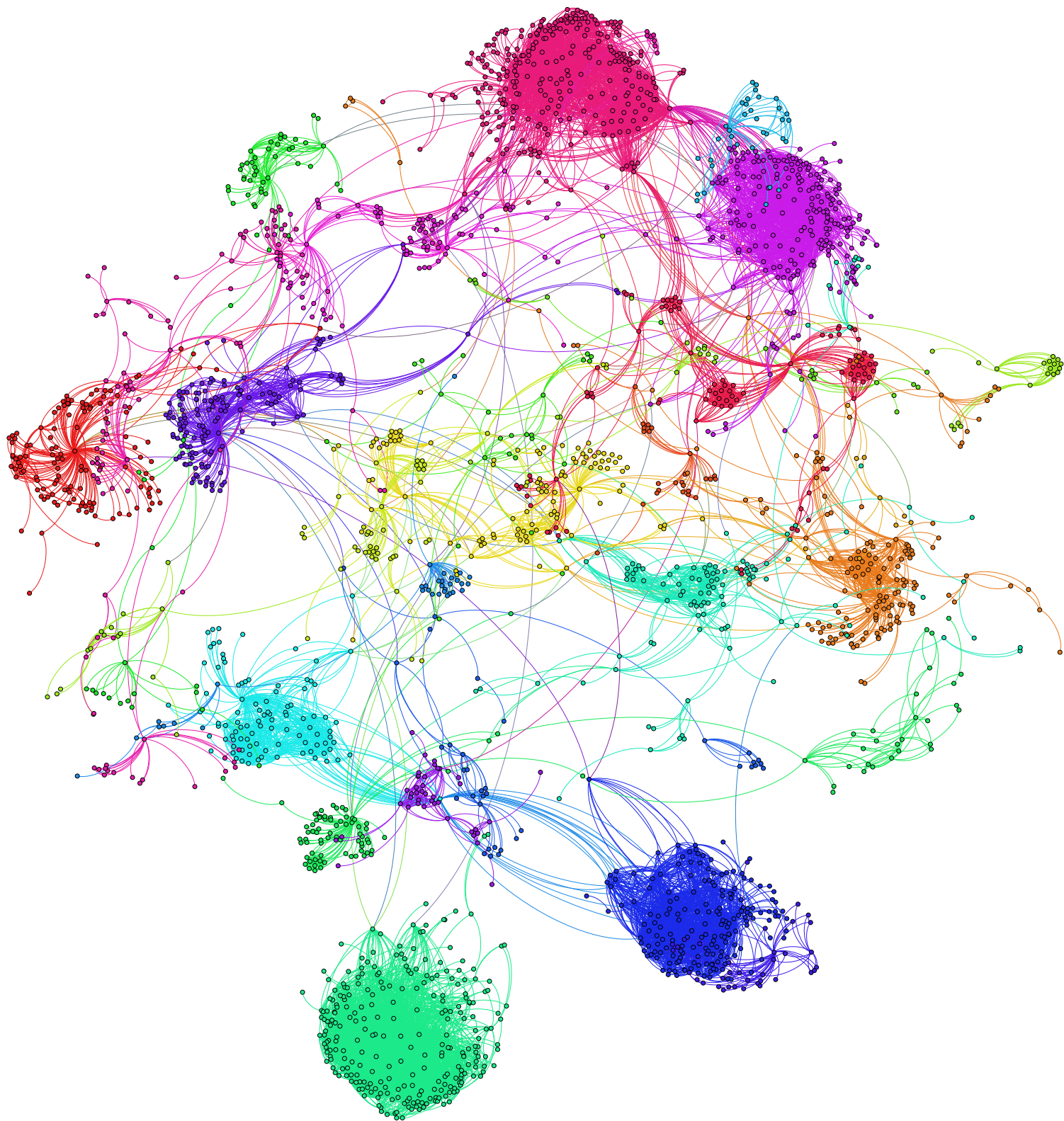

Supplement: S1 Fig — (PDF) [file pone.0128251.s003.pdf]
