# Supplementary material for: Energy Spectral Behaviors of Communication Networks of Open-Source Communities
Source: PLoS One. 2015 Jun 5;10(6):e0128251. doi: 10.1371/journal.pone.0128251 (PMC4457875; doi:10.1371/journal.pone.0128251)
Supplement: S1 File — (DOCX) [file pone.0128251.s004.docx]

**CodePlex: Terms of Use**

acceptance of terms.

The services that Microsoft provides to you are subject to the following Terms of Use ("TOU"). Microsoft reserves the right to update the TOU at any time without notice to you. The most current version of the TOU can be reviewed by clicking on the "Terms of Use" hypertext link located at the bottom of our Web pages. By accessing or using the CodePlex web site in any way, including using any Services, downloading Materials, contributing to a Project or merely browsing the CodePlex web site, you agree to and are bound by the terms of this TOU. **If you do not agree to all of the terms and conditions contained in the TOU, do not access or use the CodePlex web site.**

description of services.

Throughout this Web site, Microsoft provides you with access to a variety of resources, including download areas, communication forums and product information (collectively "Services"). The Services, including any updates, enhancements, and/or new features, are subject to the TOU.

privacy and protection of personal information.

See the [Privacy Statement](http://www.codeplex.com/site/legal/privacy) disclosures relating to the collection and use of your information.

notice specific to software available on this web site.

Any software that is made available to download from the Services ("Software") is the copyrighted work of the registered users of the Services posting the Software, or their suppliers. Use of the Software is governed by the terms of the end user license agreement, if any, which accompanies or is included with the Software ("License Agreement"). An end user will be unable to download any Software that is accompanied by or includes a License Agreement, unless he or she first agrees to the License Agreement terms. If this web site provides any Software (such as javascript) to your computer’s browser and does not include a license agreement, then Microsoft grants you the right to use that Software solely to interact through your browser with this web site. Third party scripts and code linked to or referenced from this website are licensed to you by the third parties that own such code, and are not licensed by Microsoft other than for the purpose of viewing and interacting with this website.

The Software is made available for download solely for use by end users according to the License Agreement. Any reproduction or redistribution of the Software not in accordance with the License Agreement is expressly prohibited by law.

WITHOUT LIMITING THE FOREGOING, COPYING OR REPRODUCTION OF THE SOFTWARE TO ANY OTHER SERVER OR LOCATION FOR FURTHER REPRODUCTION OR REDISTRIBUTION IS EXPRESSLY PROHIBITED, UNLESS SUCH REPRODUCTION OR REDISTRIBUTION IS EXPRESSLY PERMITTED BY THE LICENSE AGREEMENT ACCOMPANYING SUCH SOFTWARE.

THE SOFTWARE IS WARRANTED, IF AT ALL, ONLY ACCORDING TO THE TERMS OF THE LICENSE AGREEMENT. EXCEPT AS WARRANTED DIRECTLY BY MICROSOFT IN THE LICENSE AGREEMENT, MICROSOFT CORPORATION HEREBY DISCLAIMS ALL WARRANTIES AND CONDITIONS WITH REGARD TO THE SOFTWARE, INCLUDING ALL WARRANTIES AND CONDITIONS OF MERCHANTABILITY, WHETHER EXPRESS, IMPLIED OR STATUTORY, FITNESS FOR A PARTICULAR PURPOSE, TITLE AND NON-INFRINGEMENT.

FOR YOUR CONVENIENCE, MICROSOFT MAY MAKE AVAILABLE AS PART OF THE SERVICES OR IN ITS SOFTWARE PRODUCTS, TOOLS AND UTILITIES FOR USE AND/OR DOWNLOAD. MICROSOFT DOES NOT MAKE ANY ASSURANCES WITH REGARD TO THE ACCURACY OF THE RESULTS OR OUTPUT THAT DERIVES FROM SUCH USE OF ANY SUCH TOOLS AND UTILITIES. PLEASE RESPECT THE INTELLECTUAL PROPERTY RIGHTS OF OTHERS WHEN USING THE TOOLS AND UTILITIES MADE AVAILABLE ON THE SERVICES OR IN MICROSOFT SOFTWARE PRODUCTS.

YOU ACKNOWLEDGE AND AGREE THAT: (i) MICROSOFT DOES NOT CONTROL, REVIEW, REVISE, ENDORSE, OR ACCEPT RESPONSIBILITY FOR ANY MATERIALS, PROJECTS OR SERVICES OFFERED BY THIRD PARTIES, INCLUDING THIRD-PARTY VENDORS AND THIRD PARTIES ACCESSIBLE THROUGH LINKED SITES; (ii) MICROSOFT MAKES NO REPRESENTATIONS OR WARRANTIES WHATSOEVER ABOUT ANY SUCH THIRD PARTIES, THEIR MATERIALS OR SERVICES; (iii) ANY DEALINGS YOU MAY HAVE WITH SUCH THIRD PARTIES ARE AT YOUR OWN RISK; AND (iv) MICROSOFT SHALL NOT BE LIABLE OR RESPONSIBLE FOR ANY MATERIALS OR SERVICES OFFERED BY THIRD PARTIES.

notice specific to documents available on this web site.

Permission to use Documents (such as white papers, press releases, datasheets and FAQs) from the Services is granted, provided that: (1) the below copyright notice appears in all copies and that both the copyright notice and this permission notice appear, (2) use of such Documents from the Services is for informational and non-commercial or personal use only and will not be copied or posted on any network computer or broadcast in any media, and (3) no modifications of any Documents are made. Accredited educational institutions, such as K-12, universities, private/public colleges, and state community colleges, may download and reproduce the Documents for distribution in the classroom. Distribution outside the classroom requires express written permission. Use for any other purpose is expressly prohibited by law, and may result in severe civil and criminal penalties. If different or additional terms of use accompany or are included in the Documents from the Services such terms of use shall govern the use of such documents.

Documents specified above do not include the design or layout of the CodePlex.com Web site or any other Microsoft owned, operated, licensed or controlled site. Elements of Microsoft Web sites are protected by trade dress, trademark, unfair competition, and other laws and may not be copied or imitated in whole or in part. No logo, graphic, sound or image from any Microsoft Web site may be copied or retransmitted unless expressly permitted by Microsoft.

MICROSOFT AND/OR ITS RESPECTIVE SUPPLIERS MAKE NO REPRESENTATIONS ABOUT THE SUITABILITY OF THE INFORMATION CONTAINED IN THE DOCUMENTS AND RELATED GRAPHICS PUBLISHED AS PART OF THE SERVICES FOR ANY PURPOSE. ALL SUCH DOCUMENTS AND RELATED GRAPHICS ARE PROVIDED "AS IS" WITHOUT WARRANTY OF ANY KIND. MICROSOFT AND/OR ITS RESPECTIVE SUPPLIERS HEREBY DISCLAIM ALL WARRANTIES AND CONDITIONS WITH REGARD TO THIS INFORMATION, INCLUDING ALL WARRANTIES AND CONDITIONS OF MERCHANTABILITY, WHETHER EXPRESS, IMPLIED OR STATUTORY, FITNESS FOR A PARTICULAR PURPOSE, TITLE AND NON-INFRINGEMENT. IN NO EVENT SHALL MICROSOFT AND/OR ITS RESPECTIVE SUPPLIERS BE LIABLE FOR ANY SPECIAL, INDIRECT OR CONSEQUENTIAL DAMAGES OR ANY DAMAGES WHATSOEVER RESULTING FROM LOSS OF USE, DATA OR PROFITS, WHETHER IN AN ACTION OF CONTRACT, NEGLIGENCE OR OTHER TORTIOUS ACTION, ARISING OUT OF OR IN CONNECTION WITH THE USE OR PERFORMANCE OF INFORMATION AVAILABLE FROM THE SERVICES. THE DOCUMENTS AND RELATED GRAPHICS PUBLISHED ON THE SERVICES COULD INCLUDE TECHNICAL INACCURACIES OR TYPOGRAPHICAL ERRORS. CHANGES ARE PERIODICALLY ADDED TO THE INFORMATION HEREIN. MICROSOFT AND/OR ITS RESPECTIVE SUPPLIERS MAY MAKE IMPROVEMENTS AND/OR CHANGES IN THE PRODUCT(S) AND/OR THE PROGRAM(S) DESCRIBED HEREIN AT ANY TIME.

notices regarding software, documents and services available on this web site

IN NO EVENT SHALL MICROSOFT AND/OR ITS RESPECTIVE SUPPLIERS BE LIABLE FOR ANY SPECIAL, INDIRECT OR CONSEQUENTIAL DAMAGES OR ANY DAMAGES WHATSOEVER RESULTING FROM LOSS OF USE, DATA OR PROFITS, WHETHER IN AN ACTION OF CONTRACT, NEGLIGENCE OR OTHER TORTIOUS ACTION, ARISING OUT OF OR IN CONNECTION WITH THE USE OR PERFORMANCE OF SOFTWARE, DOCUMENTS, PROVISION OF OR FAILURE TO PROVIDE SERVICES, OR INFORMATION AVAILABLE FROM THE SERVICES.

member account, password, and security.

If any of the Services requires you to open an account, you must complete the registration process by providing us with current, complete and accurate information as prompted by the applicable registration form. You also will choose a password and a user name. You are entirely responsible for maintaining the confidentiality of your password and account. Furthermore, you are entirely responsible for any and all activities that occur under your account. You agree to notify Microsoft immediately of any unauthorized use of your account or any other breach of security. Microsoft will not be liable for any loss that you may incur as a result of someone else using your password or account, either with or without your knowledge. However, you could be held liable for losses incurred by Microsoft or another party due to someone else using your account or password. You may not use anyone else's account at any time, without the permission of the account holder.

no unlawful or prohibited use.

As a condition of your use of the Services, you will not use the Services for any purpose that is unlawful or prohibited by these terms, conditions, and notices. You may not use the Services in any manner that could damage, disable, overburden, or impair any Microsoft server, or the network(s) connected to any Microsoft server, or interfere with any other party's use and enjoyment of any Services. You may not attempt to gain unauthorized access to any Services, other accounts, computer systems or networks connected to any Microsoft server or to any of the Services, through hacking, password mining or any other means. You may not obtain or attempt to obtain any materials or information through any means not intentionally made available through the Services.

use of services.

The Services may contain email services, bulletin board services, chat areas, news groups, forums, communities, and/or other message or communication facilities designed to enable you to communicate with others (each a "Communication Service" and collectively "Communication Services"). You agree to use the Communication Services only to post, send and receive messages and material that are proper and, when applicable, related to the particular Communication Service. By way of example, and not as a limitation, you agree that when using the Communication Services, you will not:

Use the Communication Services in connection with surveys, contests, pyramid schemes, chain letters, junk email, spamming or any duplicative or unsolicited messages (commercial or otherwise).

Defame, abuse, harass, stalk, threaten or otherwise violate the legal rights (such as rights of privacy and publicity) of others.

Publish, post, upload, distribute or disseminate any inappropriate, profane, defamatory, obscene, indecent or unlawful topic, name, material or information.

Use any material or information, including images or photographs, which are made available through the Services in any manner that infringes any copyright, trademark, patent, trade secret, or other proprietary right of any party.

Upload files that contain viruses, Trojan horses, worms, time bombs, cancelbots, corrupted files, or any other similar software or programs that may damage the operation of another's computer or property of another.

Advertise or offer to sell or buy any goods or services for any business purpose, unless such Communication Services specifically allows such messages.

Download any file posted by another user of a Communication Service that you know, or reasonably should know, cannot be legally reproduced, displayed, performed, and/or distributed in such manner.

Falsify or delete any copyright management information, such as author attributions, legal or other proper notices or proprietary designations or labels of the origin or source of software or other material contained in a file that is uploaded.

Restrict or inhibit any other user from using and enjoying the Communication Services.

Violate any code of conduct or other guidelines which may be applicable for any particular Communication Service.

Harvest or otherwise collect information about others, including email addresses.

Violate any applicable laws or regulations.

Create a false identity for the purpose of misleading others.

Use, download or otherwise copy, or provide (whether or not for a fee) to a person or entity any directory of users of the Services or other user or usage information or any portion thereof.

Microsoft has no obligation to monitor the Communication Services. However, Microsoft reserves the right to review materials posted to the Communication Services and to remove any materials in its sole discretion. Microsoft reserves the right to terminate your access to any or all of the Communication Services at any time, without notice, for any reason whatsoever.

Microsoft reserves the right at all times to disclose any information as Microsoft deems necessary to satisfy any applicable law, regulation, legal process or governmental request, or to edit, refuse to post or to remove any information or materials, in whole or in part, in Microsoft's sole discretion.

Always use caution when giving out any personally identifiable information about yourself or your children in any Communication Services. Microsoft does not control or endorse the content, messages or information found in any Communication Services and, therefore, Microsoft specifically disclaims any liability with regard to the Communication Services and any actions resulting from your participation in any Communication Services. Managers and hosts are not authorized Microsoft spokespersons, and their views do not necessarily reflect those of Microsoft.

Materials uploaded to the Communication Services may be subject to posted limitations on usage, reproduction and/or dissemination; you are responsible for adhering to such limitations if you download the materials.

materials provided to microsoft or posted at any microsoft web site.

Microsoft does not claim ownership of the materials you provide to Microsoft (including feedback and suggestions regarding Codeplex or other Microsoft material posted on the site) or post, upload, input or submit to any Services or its associated services for review by the general public, or by the members of any public or private community, (each a "Submission" and collectively "Submissions"). However, by posting, uploading, inputting, providing or submitting ("Posting") your Submission you understand and agree that you’re giving a license under your intellectual property rights to all authorized users, including the rights to download, copy, modify, distribute and repost, unless the project licenses specify otherwise. In addition, you’re giving Microsoft all the necessary rights to make your Submission(s) available on this site and to make improvements to the CodePlex site based on your suggestions. Microsoft does not control, review, revise, endorse or distribute third-party Submissions. Microsoft is hosting the CodePlex site solely as a web storage site as a service to the developer community.

No compensation will be paid with respect to the use of your Submission, as provided herein. Microsoft is under no obligation to post or use any Submission you may provide and Microsoft may remove any Submission at any time in its sole discretion.

By Posting a Submission you warrant and represent that you own or otherwise control all of the rights to your Submission as described in these Terms of Use including, without limitation, all the rights necessary for you to provide, post, upload, input or submit the Submissions.

In addition to the warranty and representation set forth above, by Posting a Submission that contain images, photographs, pictures or that are otherwise graphical in whole or in part ("Images"), you warrant and represent that (a) you are the copyright owner of such Images, or that the copyright owner of such Images has granted you permission to use such Images or any content and/or images contained in such Images consistent with the manner and purpose of your use and as otherwise permitted by these Terms of Use and the Services, (b) you have the rights necessary to grant the licenses and sublicenses described in these Terms of Use, and (c) that each person depicted in such Images, if any, has provided consent to the use of the Images as set forth in these Terms of Use, including, by way of example, and not as a limitation, the distribution, public display and reproduction of such Images. By Posting Images, you are granting (a) to all members of your private community (for each such Images available to members of such private community), and/or (b) to the general public (for each such Images available anywhere on the Services, other than a private community), permission to use your Images in connection with the use, as permitted by these Terms of Use, of any of the Services, (including, by way of example, and not as a limitation, making prints and gift items which include such Images), and including, without limitation, a non-exclusive, world-wide, royalty-free license to: copy, distribute, transmit, publicly display, publicly perform, reproduce, edit, translate and reformat your Images without having your name attached to such Images, and the right to sublicense such rights to any supplier of the Services. The licenses granted in the preceding sentences for Images will terminate at the time you completely remove such Images from the Services, provided that, such termination shall not affect any licenses granted in connection with such Images prior to the time you completely remove such Images. No compensation will be paid with respect to the use of your Images.

notices and procedure for making claims of copyright infringement.

Pursuant to Title 17, United States Code, Section 512(c)(2), notifications of claimed copyright infringement should be sent to Service Provider's Designated Agent. ALL INQUIRIES NOT RELEVANT TO THE FOLLOWING PROCEDURE WILL NOT RECEIVE A RESPONSE. See [Notice and Procedure for Making Claims of Copyright Infringement](http://www.microsoft.com/info/cpyrtInfrg.htm).

links to third party sites.

THE LINKS IN THIS AREA WILL LET YOU LEAVE MICROSOFT'S SITE. THE LINKED SITES ARE NOT UNDER THE CONTROL OF MICROSOFT AND MICROSOFT IS NOT RESPONSIBLE FOR THE CONTENTS OF ANY LINKED SITE OR ANY LINK CONTAINED IN A LINKED SITE, OR ANY CHANGES OR UPDATES TO SUCH SITES. MICROSOFT IS NOT RESPONSIBLE FOR WEBCASTING OR ANY OTHER FORM OF TRANSMISSION RECEIVED FROM ANY LINKED SITE. MICROSOFT IS PROVIDING THESE LINKS TO YOU ONLY AS A CONVENIENCE, AND THE INCLUSION OF ANY LINK DOES NOT IMPLY ENDORSEMENT BY MICROSOFT OF THE SITE.

unsolicited idea submission policy.

MICROSOFT OR ANY OF ITS EMPLOYEES DO NOT ACCEPT OR CONSIDER UNSOLICITED IDEAS, INCLUDING IDEAS FOR NEW ADVERTISING CAMPAIGNS, NEW PROMOTIONS, NEW PRODUCTS OR TECHNOLOGIES, PROCESSES, MATERIALS, MARKETING PLANS OR NEW PRODUCT NAMES. PLEASE DO NOT SEND ANY ORIGINAL CREATIVE ARTWORK, SAMPLES, DEMOS, OR OTHER WORKS. THE SOLE PURPOSE OF THIS POLICY IS TO AVOID POTENTIAL MISUNDERSTANDINGS OR DISPUTES WHEN MICROSOFT'S PRODUCTS OR MARKETING STRATEGIES MIGHT SEEM SIMILAR TO IDEAS SUBMITTED TO MICROSOFT. SO, PLEASE DO NOT SEND YOUR UNSOLICITED IDEAS TO MICROSOFT OR ANYONE AT MICROSOFT. IF, DESPITE OUR REQUEST THAT YOU NOT SEND US YOUR IDEAS AND MATERIALS, YOU STILL SEND THEM, PLEASE UNDERSTAND THAT MICROSOFT MAKES NO ASSURANCES THAT YOUR IDEAS AND MATERIALS WILL BE TREATED AS CONFIDENTIAL OR PROPRIETARY.

copyright notice.

Copyright © 2006-2014 Microsoft Corporation, . All rights reserved.

trademarks.

Trademark information is available at [http://www.microsoft.com/mscorp/ip/trademarks/](http://www.microsoft.com/about/legal/intellectualproperty/trademarks/)

Any rights not expressly granted herein are reserved.

Send your questions to the appropriate contact as listed below:

Piracy questions can be routed to [piracy@microsoft.com](mailto:piracy@microsoft.com) or by calling 1-800-R-U-LEGIT.

General CodePlex feedback, contact us at the following page: <https://www.codeplex.com/site/contact>
